# Using Polysialylated Streptavidin as an Analytical Tool to Visualize Interaction Partners of Polysialic Acid

**DOI:** 10.3390/molecules31111928

**Published:** 2026-06-03

**Authors:** Anna Seidel, Franziska M. Kubelt, Anne Harduin-Lepers, Sebastian P. Galuska

**Affiliations:** 1 Research Institute for Farm Animal Biology (FBN), Wilhelm-Stahl-Allee 2, 18196 Dummerstorf, Germany; seidel.anna@fbn-dummerstorf.de (A.S.);; 2 Univ. Lille, CNRS, UMR 8576—UGSF—Unité de Glycobiologie Structurale et Fonctionnelle, F-59000 Lille, France; anne.harduin-lepers@univ-lille.fr

**Keywords:** polysialic acid, neutrophil extracellular traps, histones, lactoferrin

## Abstract

The interplay of the carbohydrate polysialic acid (polySia) with its interaction partners plays an important role in several physiological systems of vertebrates. The objective of this study was to develop a flexible analytical system for visualizing interaction partners of polySia using blotting and fluorescence cell staining strategies. We selected a streptavidin–biotin system due to the wide range of commercially available tools for this analytical application. After chemical polysialylation of streptavidin, the resulting bioconjugate was used to target polySia interaction partners after they had been separated via native agarose gel electrophoresis and transferred to a PVDF membrane. Furthermore, neutrophil extracellular traps (NETs) were employed to visualize polySia interaction partners within a cellular system using fluorescence-labeled biotin. The obtained results demonstrate that polysialylated streptavidin is a flexible bioconjugate that can be applied to investigate polySia interaction partners using various blotting and fluorescence imaging techniques.

## 1. Introduction

Polysialic acid (polySia) is an essential carbohydrate polymer in mammals, which has been demonstrated to mediate numerous cellular processes [1,2]. It consists of α2,8-linked *N*-acetylneuraminic acid (Neu5Ac) residues [3]. Interestingly, loss of polySia results in a lethal phenotype in a polysialyltransferase knockout mouse model [4]. Due to its polyanionic structure, polySia has the capacity to inhibit the interaction of polysialylated adhesion molecules, such as the neural cell adhesion molecule NCAM and the synaptic cell adhesion molecule SynCAM 1 [2,5,6]. The anti-adhesive capacity of polySia has been demonstrated to be involved in both physiological and pathological processes, including metastasis [3,7].

However, polySia also interacts directly with numerous peptides and proteins, thereby modulating their functions. These include growth factors, such as brain-derived neurotrophic factor (BDNF) [8], fibroblast growth factor (FGF) [9] and vascular endothelial growth factor (VEGF) [10,11]. In recent years, scientific research has revealed that several peptides and proteins within the immune system also interact with polySia. For instance, polySia interacts with lactoferrin and histones, both of which are part of antimicrobial neutrophil extracellular traps (NETs) [12]. In 2004, Brinkmann et al. discovered this novel antimicrobial meshwork consisting of extracellular DNA and several different antimicrobial molecules [13]. Histones are the most abundant proteins in NET [14] and due to their positive charge, they destroy the membrane integrity of bacteria [15]. However, histones also have a cytotoxic effect on host cells and induce an inflammatory immune response via toll-like receptors TLR-2 and TLR-4 [16]. Overall, an exaggerated NETosis and ineffective clearance can cause inflammation, leading to various severe health problems, such as sepsis, pulmonary, hepatic, nephrological or cardiovascular diseases, thrombosis, autoimmune diseases, pregnancy disorders and cancer as excellently reviewed by Islam and Takeyama [17]. Interestingly, polySia binds to the main histones of NET (H2A and H2B) and decreases their cytotoxicity against endogenous cells without inactivating their antimicrobial capacity [18,19,20]. Since soluble polySia is present in various body fluids, including blood, semen and milk, it has been hypothesized that it could act as a natural buffer against histone-mediated cytotoxicity [12]. Another interaction partner of polySia, which is present in NET, is lactoferrin, and polySia positively influences the activity of lactoferrin against an exaggerated NET formation [21]. These are only two of several examples for polySia interaction partners. In order to investigate the interaction between polySia and its potential binding partners, and to determine the biological function of this interplay, flexible tools are required to identify and visualize the target molecules of this multifunctional carbohydrate polymer.

As previously demonstrated, polySia can function as an anchor to accumulate fluorescent beads on NET by binding to its interaction partners [22,23]. However, detailed colocalization studies are limited due to the size of the particles. Furthermore, the application of polysialylated beads is restricted to cellular systems. The aim of the study was to adapt the polySia-bead strategy for use in a more flexible analytical system. The streptavidin–biotin system was chosen because of the wide range of commercially available tools for this system, including enzymes, dyes, and beads. These tools enable a wide variety of applications. We hypothesized that polysialylated streptavidin could be used as an analytical tool to visualize polySia interaction partners. We employed a range of approaches to examine the potential of polysialylated streptavidin for this purpose. To achieve this, we used known protein interaction partners, including lactoferrin and histones, and visualized them after blotting onto membranes and in NET.

## 2. Results and Discussion

### 2.1. Chemical Polysialylation of Streptavidin

**Figure 1:**
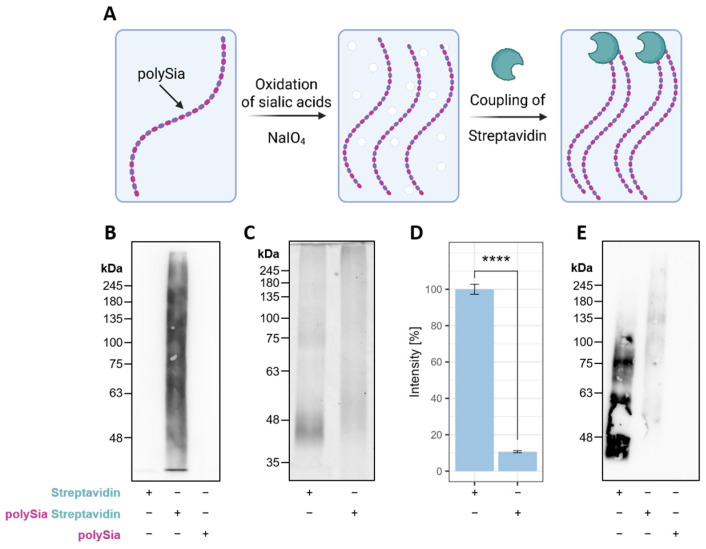
Chemical polysialylation of streptavidin. (**A**) Reaction scheme of chemical polysialylation of streptavidin. (**B**) Streptavidin, polysialylated streptavidin, and polySia were separated by 7% SDS-PAGE and transferred to a PVDF membrane. PolySia was visualized using mAb 735. (**C**) Streptavidin and polysialylated streptavidin were separated on a 10% SDS-PAGE and subsequently stained with Coomassie. (**D**) Coomassie bands between 35 and 48 kDa were quantified using ImageJ (*n* = 3). A student’s *t*-test was performed for statistical analysis. Asterisks indicate significance level (****—*p* < 0.0001). (**E**) Streptavidin, polysialylated streptavidin, and polySia were separated by 7% SDS-PAGE and stained with HRP-conjugated biotin.

In vivo, polysialylation is a well-regulated post-translational modification of glycoproteins by polysialyltransferases, resulting in only a few naturally occurring polySia carriers [24]. However, streptavidin is a bacterial protein, and the necessary *N*- and *O*-glycans for polysialylation are absent. For this reason, we employed a chemical approach for the polysialylation of streptavidin. To this end, the Neu5Ac residue at the non-reducing end was oxidized using sodium metaperiodate to introduce a chemical reactive aldehyde group (Figure 1A) [25]. The oxidized polySia chains were then incubated with a streptavidin-hydrazide conjugate. Since hydrazides react with aldehydes, the polySia strands were covalently attached to the streptavidin. The reaction products were analyzed by Western blotting after sodium dodecyl sulfate polyacrylamide gel electrophoresis (SDS-PAGE) using a monoclonal antibody (mAb) against polySia. Antibody staining of polysialylated streptavidin revealed the characteristic polySia smear [26], arising from the diversity in degree of polymerization (DP) of the polySia chains and number of chains attached to streptavidin (Figure 1B). In contrast, the negative controls, including polySia and unpolysialylated streptavidin, showed no immune signals. Thus, polySia was successfully coupled to streptavidin.

To analyze the efficiency of polysialylation, we separated the streptavidin samples before and after chemical polysialylation using SDS-PAGE and subsequently, streptavidin was visualized with Coomassie. The resulting protein stain shows that the streptavidin band between 35 and 48 kDa disappears after polysialylation (Figure 1C). Quantification revealed that approximately 90% of the streptavidin was polysialylated (Figure 1D). In contrast to the unpolysialylated streptavidin band, a broad band with lower staining intensity was visible between 48 kD and the entry point of the gel in the lane loaded with polysialylated streptavidin (Figure 1C). This indicates that polysialylated streptavidin is distributed over a wider range, which is consistent with the Western blot staining against polySia (Figure 1B). The lower signal may be due not only to the wider distribution of the polysialylated protein but also to the general inhibition of Coomassie staining of proteins by carbohydrates [27]. In line with the Western blot results, Coomassie staining demonstrates that streptavidin has been successfully polysialylated.

However, it is known that polysialylation of adhesion molecules such as Syn-CAM 1 and NCAM can inhibit homophilic interactions and the binding of antibodies to their protein part [6]. This is due to the strong negative charge of the polyanion and the resulting hydration shell. The effect depends on how close the binding domain is to the polysialylation site, and on the extent of polysialylation [2]. To investigate the impact of polysialylation on streptavidin’s ability to bind to biotin, we visualized streptavidin on polyvinylidene difluoride (PVDF) membranes using biotin-conjugated horseradish peroxidase (HRP). As shown in Figure 1C, biotin binds to both unpolysialylated and polysialylated streptavidin. As expected, polysialylated streptavidin has a higher molecular weight than unpolysialylated streptavidin. However, the observed signal intensity of polysialylated streptavidin is lower than that of unpolysialylated streptavidin. The inhibition of the homophilic interaction of NCAM and SynCAM 1 by polySia, as well as their detection by antibodies, strongly suggests that polySia also impairs the binding between biotin and streptavidin. However, the potential negative effect is not strong enough to negate the interaction. Additionally, the decrease in signal intensity may be due to a broader distribution of polySia-streptavidin on the membrane. Furthermore, the negatively charged polySia chains may affect optimal transfer to the hydrophobic PVDF membrane. It is likely that all three of these factors play a role. While polysialylation might have a negative impact on the interaction between biotin and streptavidin, the results clearly demonstrate that streptavidin can undergo chemical polysialylation without losing its biotin-binding ability in general.

**Figure 2:**
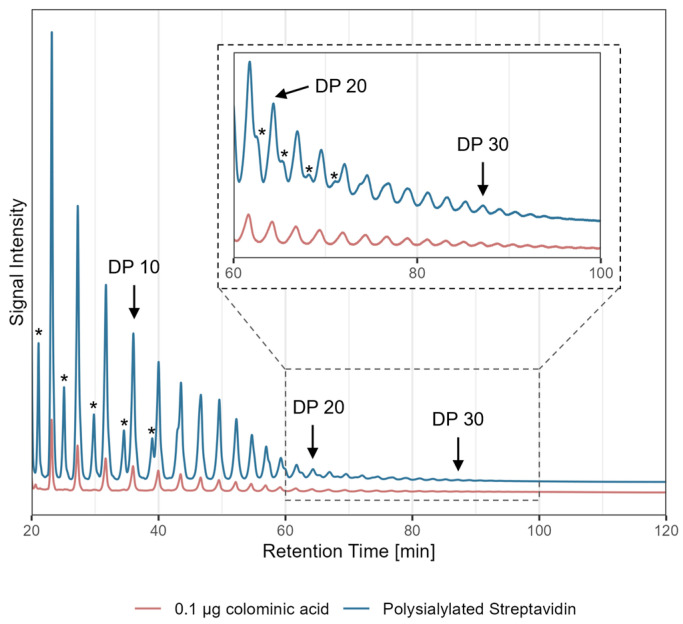
Chain length analysis of polysialylated streptavidin. Polysialylated streptavidin was precipitated using biotin magnetic beads, polySia chains were released under acidic conditions and subsequently labeled with DMB. Fluorescently labeled chains were analyzed by anion exchange chromatography (blue line). In addition, 0.1 µg colominic acid were treated and analyzed under the same conditions (red line). Chain lengths are labeled on selected peaks. Asterisks indicate additional peaks from chains containing the non-reducing ends.

Western blots against polysialylated streptavidin (Figure 1B) indicate that chains with a DP ≥ 7 must be linked to streptavidin, since the minimum chain length for mAb 735 binding is 8 [28]. However, many interaction partners require a higher DP. For instance, histones and lactoferrin require chain lengths of more than 19 for efficient binding [21,29]. To analyze the DP of the linked polySia chains, we used an HPLC fluorescence application [30]. To this end, polySia were released under acidic conditions and subsequently labeled with the fluorophore 1,2-diamino-4,5-methylenedioxybenzene (DMB). The reaction products were separated by anion-exchange chromatography according to the chain length. The results demonstrate that chain lengths are linked to streptavidin, which allow an interaction with all known interaction partners of polySia (Figure 2). The longest chains had a DP of 35. However, it is likely that considerably longer chains are present on streptavidin, given that significant internal strand breaks occur under the acidic conditions required for cleavage and labeling [31,32]. In addition to the peaks of the Neu5Ac homopolymers, peaks are visible for chains containing the non-reducing end, whose terminal Neu5Ac residues has been oxidized resulting in a seven-carbon backbone. The different retention times result from the fact that retention times are influenced by both charge and overall structure of sialic acids [33].

In summary, the results showed that streptavidin had been successfully modified with chains long enough to interact with all known interaction partners without losing its ability to bind biotin.

### 2.2. Polysialylated Streptavidin to Visualize Binding Partners of PolySia After Blotting

**Figure 3:**
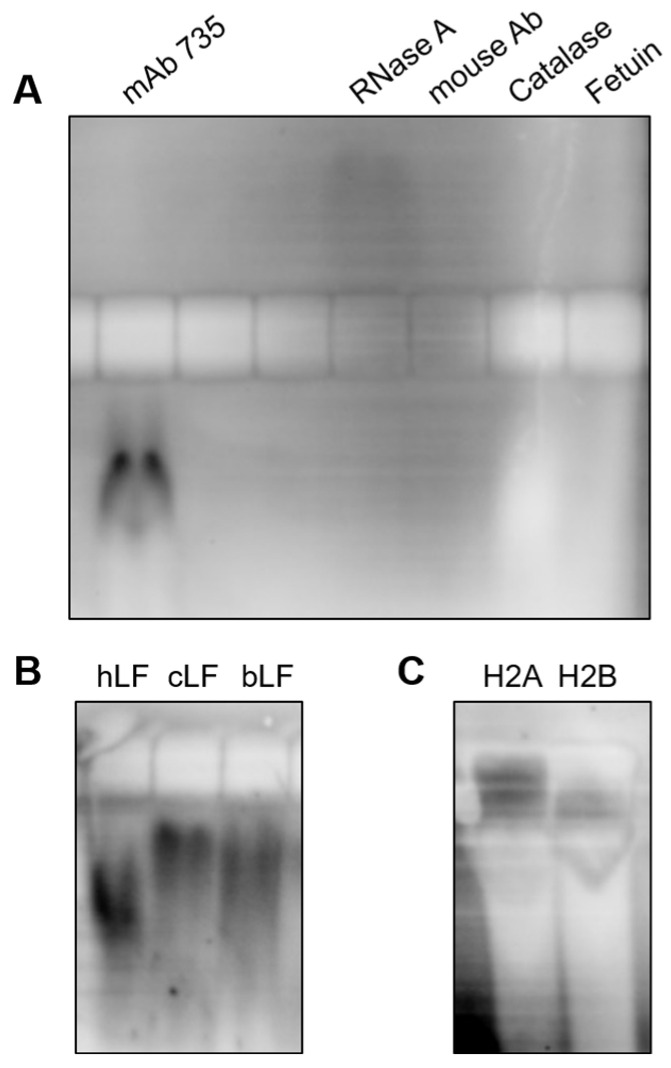
Visualization of polySia interaction partners by blotting. Native agarose gel electrophoresis was performed with (**A**) mAb 735, as well as RNase A, a mouse antibody (Ab), catalase and fetuin in addition to (**B**) lactoferrin from human milk (hLF), bovine colostrum (cLF) as well as bovine milk (bLF) and (**C**) recombinant histones H2A and H2B. Proteins were transferred onto a PVDF membrane using vacuum blotting and subsequently incubated with polysialylated streptavidin, followed by HRP-conjugated biotin.

To investigate the potential of polysialylated streptavidin to visualize interaction partners through blotting applications, we first used the mAb 735 as the target protein. Due to its exceptional binding properties, mAb 735 is probably the most commonly used mAb for Western blots against polySia. To preserve the 3-dimensional (3D) structure of the antibody, SDS-PAGE was not performed. Instead, the antibody was loaded onto a native agarose gel and then transferred to a PVDF membrane using vacuum blotting. Following a blocking step, the membrane was incubated with polysialylated streptavidin, and then with HRP-conjugated biotin to visualize the binding of the polySia chains. As shown in Figure 3A, a strong signal was observed against mAb 735, indicating that the polySia-streptavidin conjugate can visualize blotted interaction partners of polySia. As a negative control, we tested a mouse IgG, which does not target polySia, to rule out any non-specific interaction with the Fc part. Moreover, we used fetuin, catalase and RNase as negative controls. Whereas fetuin and catalase were randomly selected, RNase was used because it binds with RNA, a polyanion. As expected, no signal was observed for all four proteins.

We proceeded to test physiological binding partners of polySia, including lactoferrin [21]. Therefore, lactoferrin, which was purified from human milk, bovine colostrum and bovine milk, underwent separation via native agarose gel electrophoresis and was transferred onto a PVDF membrane. The obtained blots demonstrated that all three forms of lactoferrin were stained with polysialylated streptavidin (Figure 3B). Furthermore, we utilized the histones H2A and H2B, which are also interaction partners of polySia and the predominant histones in NETs [20]. As shown in Figure 3C, both H2A and H2B were targeted by polysialylated streptavidin, confirming the hypothesis that polysialylated streptavidin can be used to visualize polySia partners by blotting.

Overall, these experiments demonstrate that polysialylated streptavidin is a promising tool for investigating interactions between polySia and proteins, enabling efficient screening of potential interaction partners.

### 2.3. Polysialylated Streptavidin to Stain Interaction Partners in NET

**Figure 4:**
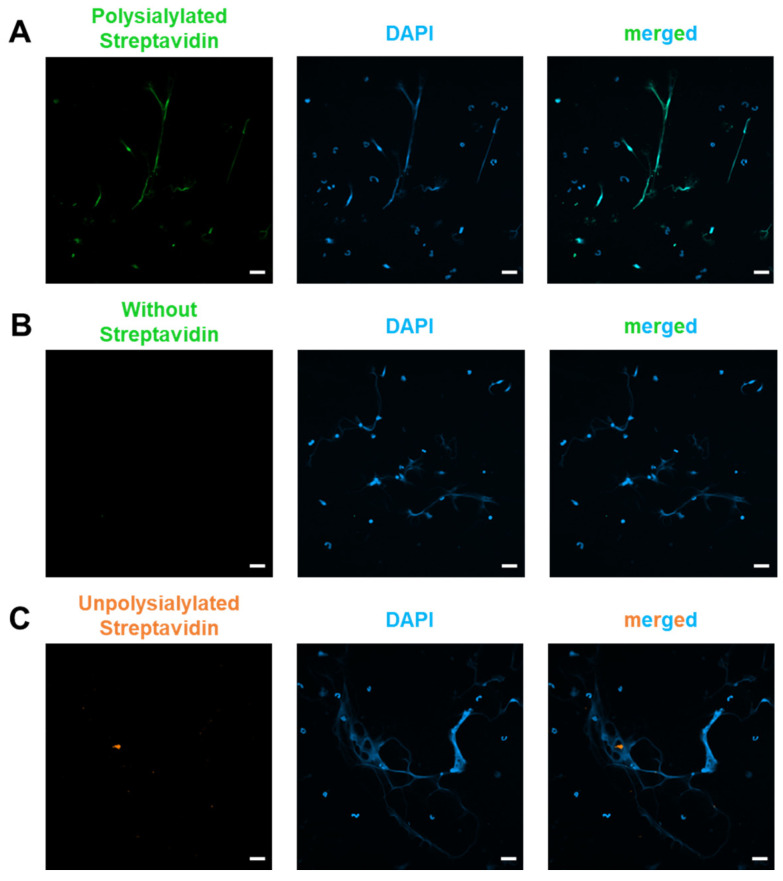
Using polysialylated streptavidin to target polySia interaction partners in NET. NETs were incubated with (**A**) polysialylated streptavidin or (**B**) without streptavidin and subsequently stained with FITC-conjugated biotin (green). (**C**) NETs were incubated with unpolysialylated streptavidin conjugated with the fluorophore AF 555 (orange). DNA was stained using DAPI (blue). Images of FITC/AF 555 signal (left column), DAPI signal (center column) and merged signal of both channels (right column) are shown. Scale bars indicate 20 µm.

It has previously been shown that polySia can accumulate fluorescent beads on NETs via interactions with histones [23]. To test the ability of polysialylated streptavidin to visualize interaction partners in NET, neutrophils were isolated from porcine blood and stimulated with lipopolysaccharide (LPS) from *Pseudomonas aeruginosa* (*P. aeruginosa*). The resulting NETs were then incubated with polysialylated streptavidin, followed by fluorescein isothiocyanate (FITC)-conjugated biotin. The deoxyribonucleic acid (DNA) was stained with 4′,6-diamidino-2-phenylindole (DAPI). Subsequent analysis using a confocal laser scanning microscope revealed an intense FITC signal that was found to be colocalized with the DAPI-stained DNA (see Figure 4A). Consequently, polysialylated streptavidin accumulates on NET fibers. FITC signal intensity increases with increasing polysialylated streptavidin concentration (Supplementary Figure S1). To verify that the binding is mediated by polySia, two negative controls were used in parallel: (1) Biotin-mediated NET staining was excluded by not adding polysialylated streptavidin (Figure 4B); (2) staining with unpolysialylated streptavidin was performed (Figure 4C). As neither of the negative controls resulted in NET staining, it was confirmed that the binding was mediated by polySia.

**Figure 5:**
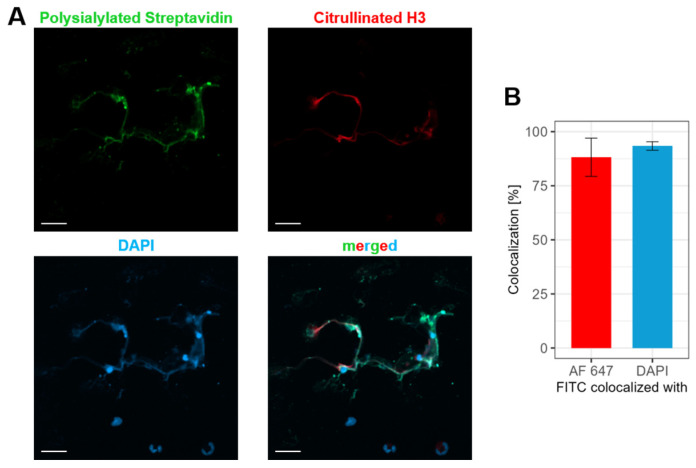
Co-staining of polySia interaction partners and citrullinated histone H3 in NETs. (**A**) NETs were first incubated with polysialylated streptavidin and an anti-citrullinated H3 antibody, followed by incubation with FITC-conjugated biotin (green) and an AF 647-conjugated anti-rabbit antibody (red). DNA was stained using DAPI (blue). Images of the FITC signal (upper left), the AF 647 signal (upper right), the DAPI signal (lower left), and the merged signal of all channels (lower right) are shown. Scale bars indicate 20 µm. (**B**) The colocalization of the FITC signal with the AF 647 signal (red) or the DAPI signal (blue) was calculated (*n* = 3 animals).

In addition, we tested whether polysialylated streptavidin could be used alongside an antibody for co-staining purposes. To this end, we employed an antibody against citrullinated histone H3 (Cit-H3), which is a marker protein for NET. As shown in Figure 5A, staining with polySia-streptavidin and anti-Cit-H3 can be performed in parallel. Both stains are located in the area of the NET filaments, which were additionally visualized with DAPI. We calculated the colocalization coefficient between the polySia-streptavidin staining and the other two stains. The data reveal correlations of 88% and 93% with Cit-H3 and DAPI, respectively (Figure 5B), confirming the presence of the described polySia interaction partners in NET. However, the interaction partner that primarily mediates the binding of polysialylated streptavidin to NET cannot be determined, as all known binding partners (histones and lactoferrin) are randomly distributed within NET [34,35].

In sum, the findings demonstrate that polysialylated streptavidin can also be used to visualize polySia interaction partners in cellular systems. However, it is also conceivable that biotinylated bioactive proteins or biotinylated beads can be easily combined with polysialylated streptavidin. For example, this could be done to enrich them on NETs. The wide variety of biotinylated proteins and beads available makes polysialylated streptavidin a versatile tool for addressing a wide range of research questions.

## 3. Materials and Methods

### 3.1. Material

Porcine blood was obtained from the Research Institute for Farm Animal Biology (FBN) slaughterhouse. Colominic acid from *Escherichia coli* (*E. coli*), an α-2,8 linked Neu5Ac homopolymer with DP 1-100 was purchased from Gerbu (Heidelberg, Germany). If not indicated otherwise, chemicals were obtained from Carl Roth GmbH (Karlsruhe, Germany). Cell culture media were obtained from Capricorn Scientific (Ebsdorfergrund, Germany).

### 3.2. Coupling of Streptavidin to PolySia

To couple polySia to streptavidin, at first polySia chains were oxidized at their terminal sialic acid residue. Therefore, 20 mg colominic acid were dissolved in 1 mL 40 mM sodium acetate buffer (pH 5.5). For the oxidation, 160 µL of 72.5 mM sodium metaperiodate, dissolved in 40 mM sodium acetate buffer, were added and the mixture was incubated in the dark on ice for 30 min. The reaction was stopped by adding 400 µL 3% ethylene glycol. To remove salts and ethylene glycol the samples were dialyzed against MilliQ water over night in a pre-treated regenerated cellulose (RC)-tubing (Molecular weight cut-off (MWCO): 1 kDa, Spectrum Labs, New Brunswick, NJ, USA) and the samples were subsequently dried in a vacuum concentrator. For coupling, 10 mg of oxidized polySia were mixed with 2 mg Streptavidin hydrazide (Thermo Scientific, Rockford, IL, USA) and incubated for 2 h at room temperature. Aliquots of polysialylated streptavidin were stored at −20 °C. Polysialylated streptavidin can be stored at −20 °C for at least six months. Longer periods were not tested.

### 3.3. SDS-PAGE & Western Blot

Samples were separated using a 7% SDS-PAGE under reducing conditions. For Western blotting, the separated proteins were transferred onto a PVDF membrane. To detect polysialylated proteins, antibody staining was performed using 1 µg/mL mAb 735 (Hannover Medical School (MHH), Hanover, Germany) as primary and 0.13 µg/mL HRP-conjugated donkey anti-mouse (Jackson Immunoresearch, Ely, UK) as secondary antibody. Moreover, membranes were stained with 1 µg/mL Biotin-HRP. For the detection of chemiluminescence signals, the membrane was incubated with ECL (8 mL 0.25 mg/mL Luminol in 100 mM Tris, HCl, pH 8.6, 3.2 µL 30% H_2_O_2_, 0.8 mL 1.1 mg/mL Coumaric acid in dimethylsulfoxide (DMSO)) and imaging was performed using the ChemiDoc MP imaging system (BioRad, Feldkirchen, Germany). All experiments were repeated at least three times.

### 3.4. SDS-PAGE & Coomassie

Samples were separated using 10% SDS-PAGE under reducing conditions. Subsequently, the samples were stained with RotiBlue Coomassie staining solution overnight at room temperature. The gels were then destained with Milli-Q water and imaged using the ChemiDoc MP system (Bio-Rad, Feldkirchen, Germany). ImageJ (v1.54g) was used to quantify the protein bands (*n* = 3) [36].

### 3.5. Chain Length Analysis

To analyze the DP of polySia covalently coupled to streptavidin, the polysialylated streptavidin was precipitated using biotin-coupled magnetic beads (Ray Biotech, Peachtree Corners, GA, USA). The polySia chains were then released via hydrolysis using 20 mM trifluoroacetic acid at 55 °C for 2 h. In parallel, 0.1 µg of colominic acid and a blank control without polySia were treated under the same conditions. After hydrolysis, the supernatant from the beads, the colominic acid standard, and the blank control were dried in a vacuum concentrator. Fluorescence labeling using DMB and subsequent analysis by anion-exchange HPLC were performed as recently described [33]. The chain length analysis was repeated three times.

### 3.6. Native Agarose Gel Electrophoresis and Vacuum Blotting

The proteins were prepared in a final volume of 12 µL of 1× Tris-buffered saline (TBS; pH 7.4) and 1 µL of glycerol was added to each sample. The samples were loaded onto a 0.7% agarose gel prepared with running buffer (25 mM Tris-HCl and 19.2 mM glycine at pH 8.5). Electrophoresis was carried out at 80 V in an electrophoresis chamber filled with running buffer. The gel was then equilibrated in blotting buffer (0.6 M NaCl, 60 mM Na_2_Citrate, pH 7.0) for 20 min. A PVDF membrane was soaked in methanol, rinsed once with water, and stored in blotting buffer. For vacuum blotting, the porous membrane was soaked in water and placed in the blotting chamber. The PVDF membrane was placed on top, followed by a plastic film with a cutout the size of the membrane. Finally, the agarose gel was placed on top, and a vacuum of 55 mbar was applied. The gel was wetted with blotting buffer every 15 min, and blotting was conducted for 2 h. Subsequently, the membrane was blocked with 5% skim milk in TBS containing 0.1% Tween (TBS-T) for 1 h at room temperature. The membrane was then washed three times for 10 min with TBS-T. The membrane was then incubated overnight at 4 °C with 5 µg/mL polysialylated streptavidin, followed by incubation with 1 µg/mL HRP-conjugated biotin for 45 min at room temperature. Chemiluminescence signals were detected using ECL solution with the ChemiDoc MP imager (BioRad, Feldkirchen, Germany). All experiments were repeated at least three times.

### 3.7. Isolation of Neutrophil Granulocytes

Porcine neutrophils were isolated using a protocol modified from Schuberth et al. [37]. Therefore, 6 mL of ethylenediaminetetraacetic acid (EDTA) whole blood were mixed with 6 mL sterile Dulbecco’s phosphate-buffered saline (DPBS) and gently poured over 6 mL Histopaque 1077. After density gradient centrifugation at 1275× *g* and 10 °C for 30 min without break, neutrophil granulocytes accumulated in the pellet together with the erythrocytes. The supernatant was discarded and the erythrocytes were hypotonically lysed by resuspending the pellet in 10 mL sterile MilliQ water. After 10 s 10 mL of 2× concentrated phosphate-buffered saline (PBS) were added to restore isotonic conditions and the mixture was centrifuged at 500× *g* and 4 °C for 10 min. After discarding the supernatant, the lysis step was repeated as described, except that centrifugation was carried out with only 220× *g*. After discarding the supernatant, the neutrophil granulocyte pellet was washed two times by resuspending it in 2 mL DPBS and centrifugation at 220× *g* for 5 min. Thereafter, 3 × 10^4^ cells were seeded in a total volume of 100 µL Roswell Park Memorial Institute (RPMI) minimal medium in each well of a 12-well chamber slide (Ibidi, Gräfelfing, Germany). Neutrophils were incubated for 1 h at 37 °C and 5% CO_2_ for recovery. Subsequently, NETosis was induced by adding LPS from *P. aeruginosa* in a final concentration of 50 µg/mL to the cells and incubated at 37 °C and 5% CO_2_ for 2 h. To stop the NET induction and fix the cells onto the glass slide, paraformaldehyde (PFA) was added to a final concentration of 4% and incubated at 4 °C for 20 min. After fixation, the wells were washed three times with PBS.

### 3.8. Visualization of NETs Using Streptavidin-PolySia

To perform the NET staining, at first, non-specific protein binding sites were blocked by adding 2% immunoglobulin G (IgG)-free bovine serum albumin (BSA) in PBS to the wells and incubating at 37 °C for 2 h. Then, the wells were incubated with different concentrations of polysialylated streptavidin PBS containing 0.1% BSA overnight at 4 °C. After washing the wells three times with PBS, a final concentration of 0.5 µg/mL FITC-conjugated biotin in PBS containing 0.1% BSA was incubated on the wells for 30 min. For staining with unpolysialylated streptavidin, after blocking, the wells were incubated overnight with 100 µg/mL AF 555-conjugated streptavidin (Molecular Probes, Eugene, OR, USA) in PBS with 0.1% BSA. For co-staining of polysialylated streptavidin and Cit-H3, the wells were incubated with 100 µg/mL polysialylated streptavidin and 3.33 µg/mL rabbit anti-Cit-H3 antibody (abcam, Cambridge, UK) in PBS containing 0.1% BSA, followed by 3 washing steps and incubation with 0.5 µg/mL FITC-conjugated biotin and 10 µg/mL AF 647-conjugated goat anti-rabbit antibody (Thermo Fisher Scientific, Waltham, MA, USA). The wells were washed three times with PBS before staining with 5 µM DAPI in PBS for 5 min. After three washing steps, the wells were incubated with 2% PFA at room temperature for 20 min to enhance the appearance of the fluorescent dyes. Finally, the slides were mounted using Kaiser’s glycerol gelatin and fluorescence images were taken using the confocal laser scanning microscope LSM 800 (Zeiss, Oberkochen, Germany). The colocalization of FITC signal with AF 647 and DAPI was analyzed using ImageJ (v1.54g) and the JACoP plugin [38]. The Pearson coefficient of FITC overlapping AF 647 or DAPI using the Costes‘ automatic threshold was calculated for 3 individual images.

### 3.9. Statistical Analysis and Data Visualization

For statistical analysis a Student’s *t*-test was performed using R (v4.5.2) [39]. All plots and chromatograms were created using R (v4.5.2), the readxl package (v1.4.5) [40], the tidyverse package (v2.0.0) [41] and the ggsignif package (v0.6.4) [42]. Figure 1A and the graphical abstract were created using BioRender.com.

## 4. Conclusions

The results of the outlined experiments demonstrate that polysialylated streptavidin can be used to investigate the interaction partners of polySia through blotting and cell staining. The wide variety of commercially available biotinylated tools, including enzymes, dyes and beads, allows for a diverse range of applications in detecting interaction partners within various systems and combinations. Consequently, in addition to conventional applications such as gel filtration and native gel electrophoresis, which are often used to characterize interaction with polySia, polysialylated streptavidin is a new, flexible analytical tool for visualizing polySia interaction partners using various analytical strategies.

## Data Availability

The original contributions presented in this study are included in the article/Supplementary Materials. Further inquiries can be directed to the corresponding author.

## Supplementary Materials

The following supporting information can be downloaded at https://www.mdpi.com/article/10.3390/molecules31111928/s1, Figure S1: Concentration-dependent binding of polysialylated-streptavidin on NET.

## Abbreviations

The following abbreviations are used in this manuscript:

3D	3-dimensional
AF	Alexa Fluor
BDNF	Brain-derived neurotrophic factor
BSA	Bovine serum albumin
DNA	Deoxyribonucleic acid
DAPI	4′,6-Diamidin-2-phenylindol
DMSO	Dimethyl sulfoxide
DP	Degree of polymerization
DPBS	Dulbecco’s phosphate-buffered saline
*Escherichia coli*	*E. coli*
EDTA	Ethylenediaminetetraacetic acid
FBN	Research Institute for Farm Animal Biology
FGF	Fibroblast growth factor
FITC	Fluorescein isothiocyanate
HRP	Horse-radish peroxidase
IgG	Immunoglobulin G
LPS	Lipopolysaccharide
mAb	Monoclonal antibody
MHH	Hannover Medical School
MWCO	Molecular weight cut-off
NET	Neutrophil extracellular trap
Neu5Ac	*N*-acetylneuraminic acid
PBS	Phosphate-buffered saline
*P. aeruginosa*	*Pseudomonas aeruginosa*
polySia	Polysialic acid
PVDF	Polyvinylidene difluoride
RC	Regenerated cellulose
RPMI	Roswell Park Memorial Institute
SDS-PAGE	Sodium dodecyl sulfate polyacrylamide gel electrophoresis
TBS	Tris-buffered saline
TBS-T	Tris-buffered saline containing 0.1% Tween-20
TLR	Toll-like receptor
VEGF	Vascular endothelial growth factor
