# Supplementary material for: Using Polysialylated Streptavidin as an Analytical Tool to Visualize Interaction Partners of Polysialic Acid
Source: Molecules. 2026 Jun 3;31(11):1928. doi: 10.3390/molecules31111928 (PMC13258239; doi:10.3390/molecules31111928)
Supplement: Supplementary file 1 [file molecules-31-01928-s001.zip › Seidel et al_supplementary information.pdf]

molecules

Article

Using Polysialylated Streptavidin as an Analytical Tool to Visualize Interaction Partners of Polysialic Acid

Anna Seidel [1], Franziska M. Kubelt [1], Anne Harduin-Lepers [2] and Sebastian P. Galuska [1,*]

[1] Research Institute for Farm Animal Biology (FBN), Wilhelm-Stahl-Allee 2, 18196 Dummerstorf, Germany; seidel.anna@fbn-dummerstorf.de (A.S.)
[2] Univ. Lille, CNRS, UMR 8576 - UGSF - Unité de Glycobiologie Structurale et Fonctionnelle, F-59000 Lille, France; anne.harduin-lepers@univ-lille.fr
* Correspondence: galuska.sebastian@fbn-dummerstorf.de; Tel.: +49-38208 68-769

Supplementary Information

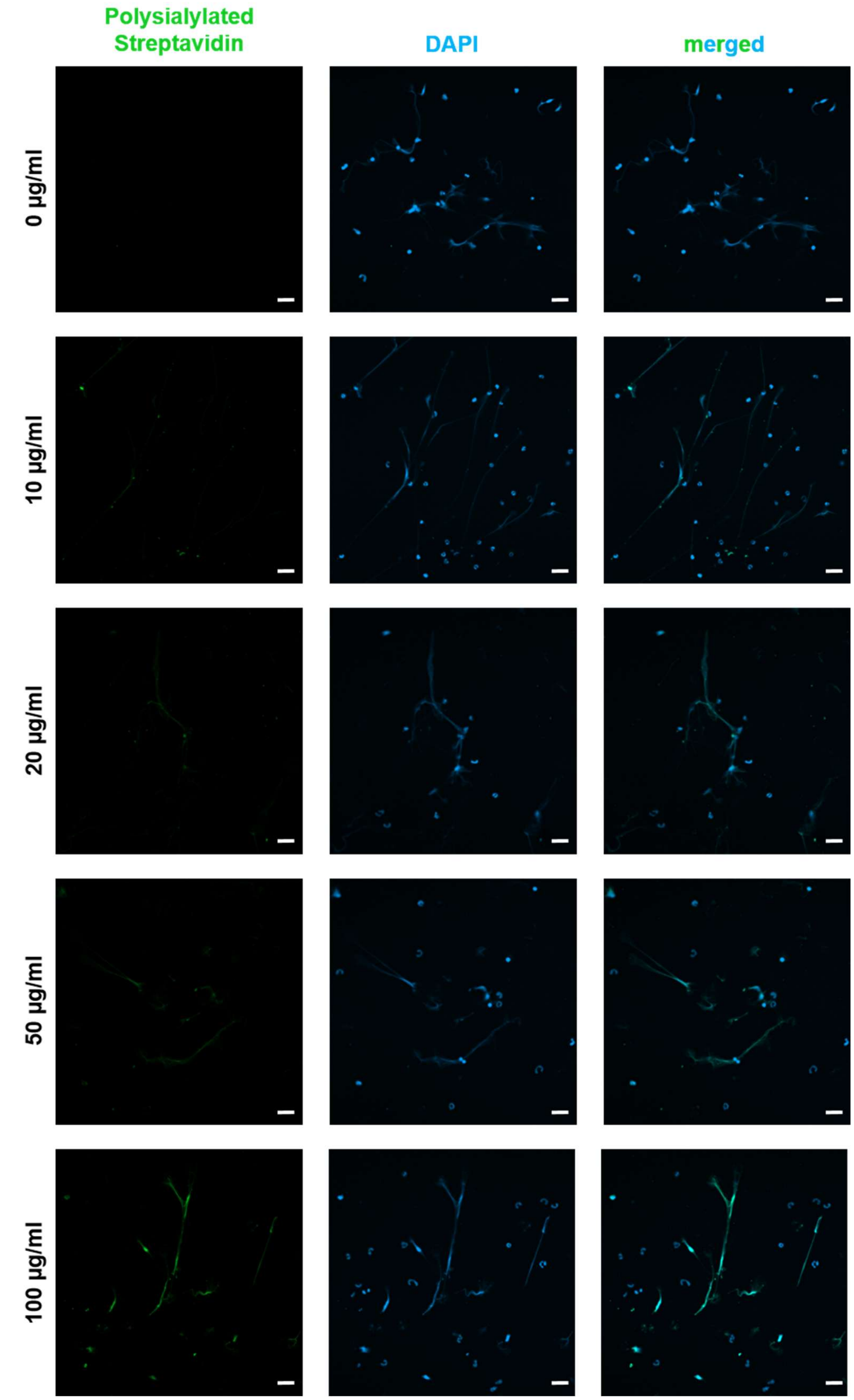

Figure S1. Concentration-dependent binding of polysialylated-streptavidin on NET. NETs were incubated with different concentrations of polysialylated streptavidin, indicated on the left. Subsequently, staining with FITC-conjugated biotin was performed. DNA was visualized using DAPI. Fluorescence signals were detected using a confocal LSM 800 (Zeiss) with a 40 × oil objective. Images of FITC signal (left column), DAPI signal (center column) and merged signal of both channels (right column) are shown. Scale bars indicate 20 µm. Pictures of 0 µg/ml and 100 µg/ml are also displayed in Figure 3.
